# In Situ, Real‐Time Imaging of pH Gradients and Solution Flows through Silica Garden Membranes during Tube Growth

**DOI:** 10.1002/smll.202507110

**Published:** 2025-12-22

**Authors:** Arianna Menichetti, Jeannette Manzi, Dario Mordini, Fermín Otálora, Juan Manuel García‐Ruiz, Marco Montalti

**Affiliations:** ^1^ Department of Chemistry “Giacomo Ciamician” Bologna Italy; ^2^ Instituto Andaluz de Ciencias de la Tierra Consejo Superior de Investigaciones Científicas Armilla Spain; ^3^ Donostia International Physics Center Donostia/San Sebastián Spain

**Keywords:** fluorescence imaging, pattern formation, self‐organization, silica gardens

## Abstract

Silica gardens are self‐organized structures formed by the reaction between high‐alkaline silicate and acidic metal solutions. This reaction generates complex tubular structures driven by osmosis and buoyancy, which are decorated by the precipitation of a diaphragm‐like membrane featuring textural and chemical gradients. It is understood that the initial gradient of pH values and its evolution over time and space are the key parameters controlling textural and chemical patterns and morphogenesis. We have developed a technique that enables simultaneous visualization of membrane formation, fluid and particle flow, and pH evolution at microscopic resolution in real‐time. This is achieved by exploiting the unusual behavior of pH probes in the saturated metal solution, which we have investigated in detail and utilized to perform ratiometric pH mapping in situ and in real‐time, either at low magnification with a color camera or, at high magnification, with a confocal microscope. Using the same probes and setup, confocal fluorescence microscopy allows for mapping pH in the microtubes with higher resolution through ratiometric analysis.

## Graphical abstract

A fluorescent pH probe, added to a saturated metal ion solution, allows for mapping pH during the growth of a silica garden. The probe shows a very intense blue fluorescence at pH around 4 and an intense green fluorescence at pH above 6. The red component of the image allows detection of the formation of particles and tracking solution flows in the silica garden.

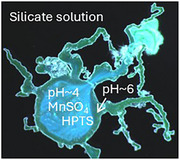

## 1 Introduction

Abiotic mineral self‐organization is an important research area for understanding pattern formation in Materials and Earth Sciences [1, 2, 3]. Many of these patterns form under far‐from‐equilibrium conditions, leading to complex hierarchical structures. Among them, silica‐induced carbonate biomorphs and silica gardens are the subjects of increased interest because they have applications in various scientific and technological fields, from catalysis [4] to functional advanced materials [5, 6, 7, 8] to terrestrial and extraterrestrial geochemistry [9, 10, 11], and primitive life detection [12]. Silica gardens are tubular, diaphragm‐like membranes formed upon the reaction of highly alkaline sodium silicate solutions with acidic solutions of soluble metal salts. In many cases, the membranes are made of amorphous silica and metal oxy‐hydroxide nanoparticles exhibiting chemical and textural gradients, although carbonate membranes can also be formed in other chemical gardens [13, 14]. They form by the far‐from‐equilibrium chemical precipitation of fluid structures driven by osmosis and buoyancy. Interestingly, it has been experimentally demonstrated that the chemical and diffusion potential across the membrane generates a voltage of hundreds of mV between the inner and outer solutions [15], which converts this type of structure into microreactors for catalytic studies [16]. This property of silica gardens has been tested in organic synthesis and the origin of life studies [17]. It has been shown that micron‐sized vesicles formed by mixing microdrops of a solution of metallic salts with a solution of sodium silicate are excellent catalysts for the condensation of formamide, and that the catalysis is selective depending on the silica‐rich or metal‐rich sides of the membrane [18, 19].

Nevertheless, in‐situ real‐time investigation of the growth of silica garden is still very limited, and therefore, the detailed mechanisms of morphogenesis and patterning are still obscure [20, 21, 22, 23, 24, 25, 26, 27, 28].

It is well‐known that, in silica gardens, the initial pH gradient between the two reactive solutions and its temporal and spatial evolution are key parameters that control morphogenesis and microstructural patterning. So far, pH values and other chemical variables have only been monitored on a large scale [14, 16, 17, 29, 30]. This is primarily due to the difficulty of applying conventional imaging techniques, such as fluorescence microscopy, to these systems [31, 32]. Fluorescence imaging is a powerful technique [33, 34] widely used to measure chemical parameters like local pH by utilizing fluorescent molecular probes incorporated as contrast agents in these systems [32]. The efficiency of this imaging technique has been widely demonstrated in biology and related life sciences, both for application in vitro and in vivo [35, 36, 37, 38].

Nevertheless, developing fluorescent probes suitable for application in very concentrated metal solutions, such as those involved in synthesizing silica gardens, is still challenging and largely unexplored. Metal ions are indeed well‐recognized fluorescence quenchers and typically interfere with the probe response, altering the detection and making pH measurement strongly dependent on local chemical properties different from hydronium ion concentration [39]. Here, we demonstrate that a well‐known pH‐sensitive fluorescent probe 8‐hydroxypyrene‐1,3,6‐trisulfonic acid (HPTS) shows, in saturated metal solutions, a very peculiar response to pH completely different from what is reported for aqueous solution [40, 41]. In particular, the probe shows a different pH dependency of the fluorescence in the blue and the green regions, and the two signals can be combined to produce ratiometric pH detection, which is essential for measuring pH in spatially inhomogeneous and time‐evolving systems like silica gardens [42, 43]. For the experiment, we have developed a design inspired by the reactor used in the experiment on the condensation of formamide [4]. The quasi‐2D silica gardens were first investigated, as a whole, using a dedicated setup for wide‐field fluorescence imaging and then locally by analyzing the microtubes with fluorescence laser confocal scanning microscopy (LCSM). By exploiting the properties of the pH fluorescent probe HPTS, which emits very intense, blue and green light in a pH‐dependent manner, the fluorescence could be detected using a conventional CMOS color (RGB) camera, using the G and B channels for ratiometric pH measurement and reserving the red channel for morphological and pH‐independent visualization of the membrane structure (see section 2.2 for details). In this approach, time‐lapse images could also be used to observe the spontaneous formation of microparticles, which could be tracked to visualize the solution flows. Eventually, LCSM, which guarantees sub‐micron resolution along the z‐axis, was employed to map the temporal pH distribution inside the microtubes.

## 2 Results and Discussion

### 2.1 The Silica Garden Growth Experiment

**FIGURE 1:**
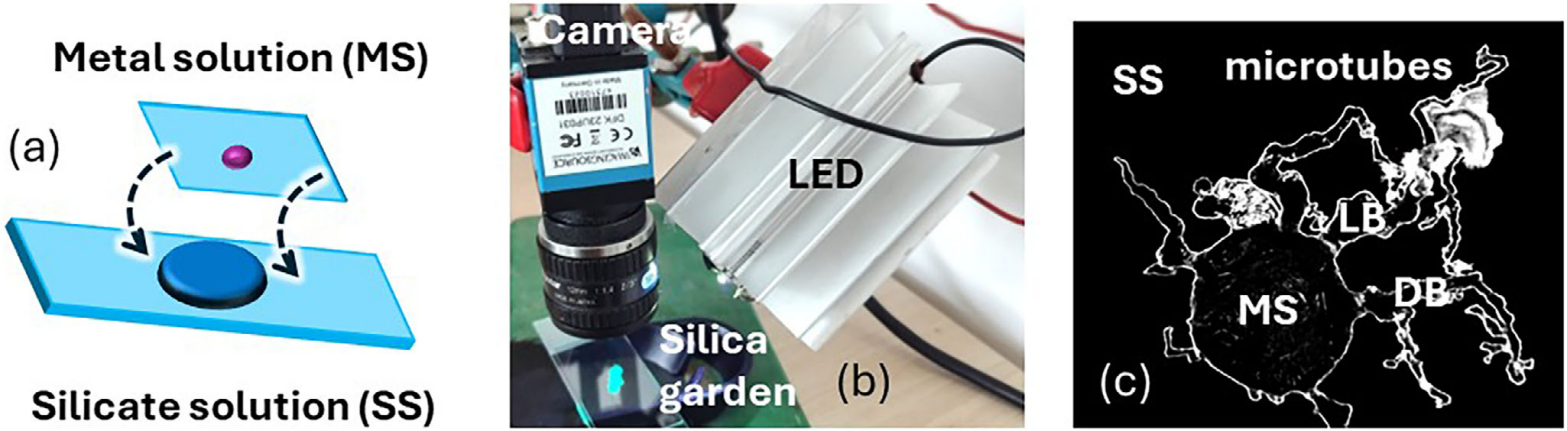
Left: preparation of the silica garden: a 2 µl droplet of saturated MnSO_4_, metal solution (MS), is immersed in 50 µl of silicate solution (SS). Right: a circular membrane is formed at the interface of the two solutions, and some branches immediately erupt from this membrane. Growth of microtubes is observed only from one branch (living branch, LB) but not from the others (dead branches, DB).

The procedure followed for the preparation of the silica gardens is schematized in Figure 1: a small drop (2 µL) of a saturated solution of the metal salt, MnSO_4_ (metal solution, MS), was deposited in the center of a thin glass coverslip. Simultaneously, a larger volume of the silicate solution (50 µL, SS) was dropped onto the top of a thicker and larger glass slide. The glass coverslip with the manganese solution was gently turned upside down and brought on top of the silicate solution. For locally measuring the pH, the fluorescent probe HPTS was added at a final concentration of 0.1 mM to the Mn solution. We would like to stress that our approach was optimized for Mn^2+^ ions and it cannot be extended (without proper redesign) to other metal ions.

The final result was a sideways open glass cell with the Mn solution roughly confined in the center of the silicate solution. The fast formation of an almost circular membrane at the interface between the two solutions prevented mixing, and the growth of tubes in the horizontal plane could be observed after glass cell closure. As shown in Figure 1, some branches are already formed at the beginning of the observation (about 5 seconds after cell closure). Some of these branches grow in time, producing new branches and microtubes (living branches, LB in Figure 1). Other branches do not grow during the experiment (dead branches, DB in Figure 1), nevertheless, as it will be shown, they are involved in the overall evolution of the silica garden.

### 2.2 Imaging pH in Real Time in Silica Gardens

**FIGURE 2:**
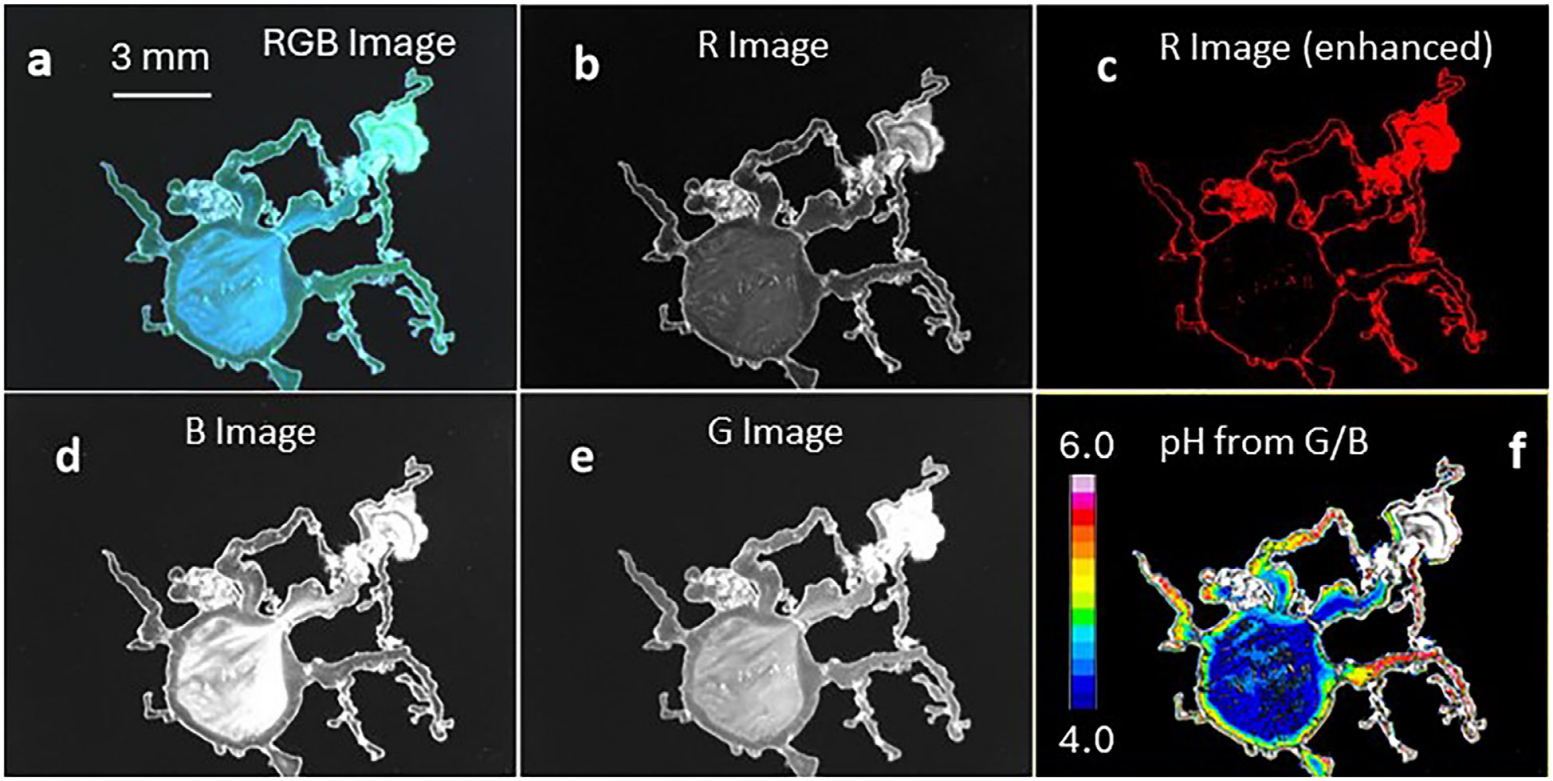
(a) First image of the silica garden acquired with an RGB camera, blue‐green coloration is due to fluorescence of the pH probe, and it is pH dependent. b) red component of the image, this is largely pH independent and shows the morphology of the membrane. Contrast of the red image was enhanced with ImageJ to get a better‐defined membrane profile, as shown in c. The blue and green components are shown in d and e, respectively, and they were used to calculate the intensity ratio between the green and red components and hence the pH map, shown in f.

The setup we used for imaging pH in the growing silica garden is shown in Figure 1b. Illumination is based on a focused UV LED, and a color CMOS camera is used for time‐lapse image acquisition in reflection mode (both light source and the camera are on the same side with respect to the sample). Using this setup, the whole silica garden could be observed with the camera. The color camera detects independently the red (R), green (G), and blue (B) components of the image (see Supporting Information for details). The LED spectrum (see Supporting Information) presents a strong peak in the UV region (366 nm), which is not detected by the camera, and a weak residual emission in the visible. Thanks to this spectral profile, LED light can be used to excite the pH probe (HPTS) added to the metal salt solution, producing very intense fluorescence in the blue and in the green regions with a pH‐dependent relative intensity (fluorescence in the red is negligible, see Supporting Information), but also as a weak white light source. This weak white light emitted by the LED is scattered by the inorganic membrane constituting the “walls” of the silica garden, allowing their imaging. Importantly, in the chosen experimental conditions, i) B and G scattered light components are negligible with respect to the B and G light emitted by the pH probe, and ii) the R component of fluorescence is negligible with respect to the R scattered light. Hence, the B and G components measure only fluorescence intensity, while the R component measures only scattering intensity. This means that when the color image is split into the R, G, and B components, R shows only the “structure” of the silica garden while G and B show the local fluorescence coming from the solutions. The information achievable is shown in Figure 2.

### 2.3 Initial Properties of the Silica Gardens

The imaging of the silica gardens started immediately after the preparation. Nevertheless, the closure of the optical cell and the positioning on the optical acquisition stage required about five seconds. In the next discussion, we refer to this time as the initial one (0 seconds in the time scale). The starting RGB color image acquired in a typical experiment is shown in Figure 2a. A circular membrane is immediately formed at the interface between the metal and the silicate solution. Interestingly, Figure 2a shows that the metal solution presents strong blue and green fluorescence, while the non‐fluorescent silicate solution appears as a dark background. We would like to stress that in blank experiments, hence performed in the same conditions in the absence of the pH probe in the metal solution, no fluorescence could be detected from the metal solution itself.

In Figure 2a, it is possible to localize the starting metal solution droplet and to observe that, because of the compression occurring during the cell preparation, a small part of the metal solution is ejected into the silicate, producing some branches. In Figure 2a, it can be easily observed that the fluorescence color greatly changes in different regions of the silica garden. In particular, it can be clearly observed that the fluorescence is blue‐cyan in the inner part, while it is green in the proximity of the membrane. In order to correlate the color of the fluorescence to pH, we performed a calibration experiment by observing MS, containing HPTS 0.1 mM with controlled pH, as discussed in the experimental section.

Detailed information can be extracted from color images by splitting the image into the three red, green, and blue (RGB) components. In particular, the R component corresponds to wavelength λ > 600 nm. Hence, the fluorescence spectra of the pH probe, shown in the SI, clearly confirm that no fluorescence can be detected on the red channel independently of the pH. Thanks to this feature, as mentioned, the red channel can be used to visualize the morphology and follow its growth. Figure 2b indeed shows the red component of the image in Figure 2a, the membrane of the silica garden can be detected, but not the fluorescence from the MS. The contrast can be further enhanced by processing the image as in Figure 2c. The blue B (Figure 2d) and the green component G (Figure 2e) are pH dependent and can be combined in a ratiometric approach (see Supporting Information) to obtain the actual pH, as shown in Figure 2f. We would like to stress that while the use of molecular probes presents a unique advantage, high spatial and temporal resolution in pH detection, the actual pH windows which is normally detectable with this kind of probe are of two units, pK_a_±1.0, where pK_a_ is characteristic of the probe. Hence, in this work, we will analyze pH in the 4.0–6.0 pH interval.

Figure 2a clearly shows that at the beginning of the experiment, the pH in the inner part of the metal solution is the same as the starting solution, while in the proximity of the membrane, the pH is considerably higher. This demonstrates that during or immediately after the formation of the membrane, alkalinization of the MS occurs because of the permeation of OH^−^ ions through the membrane. The thickness of this band of less acidic solution can be easily measured by analyzing the profile of the fluorescence perpendicularly to the membrane, and it is about 0.5 mm. The formation of this less acidic band is due to the moderate permeability of the initially formed membrane, because of which OH^−^ ions can diffuse through the membrane, causing a pH increase. This process, on the other hand, causes the acidification of the silica solution in close proximity to the outer surface of the membrane, producing, as will be discussed in the following sections, silica deposition and a gradual decrease in the permeability. Although in this paper we discuss the results for a specific silica garden, the experiment was repeated several times and results for five different silica gardens are reported in the SI.

### 2.4 Imaging the Silica Gardens during Their Growth

**FIGURE 3:**
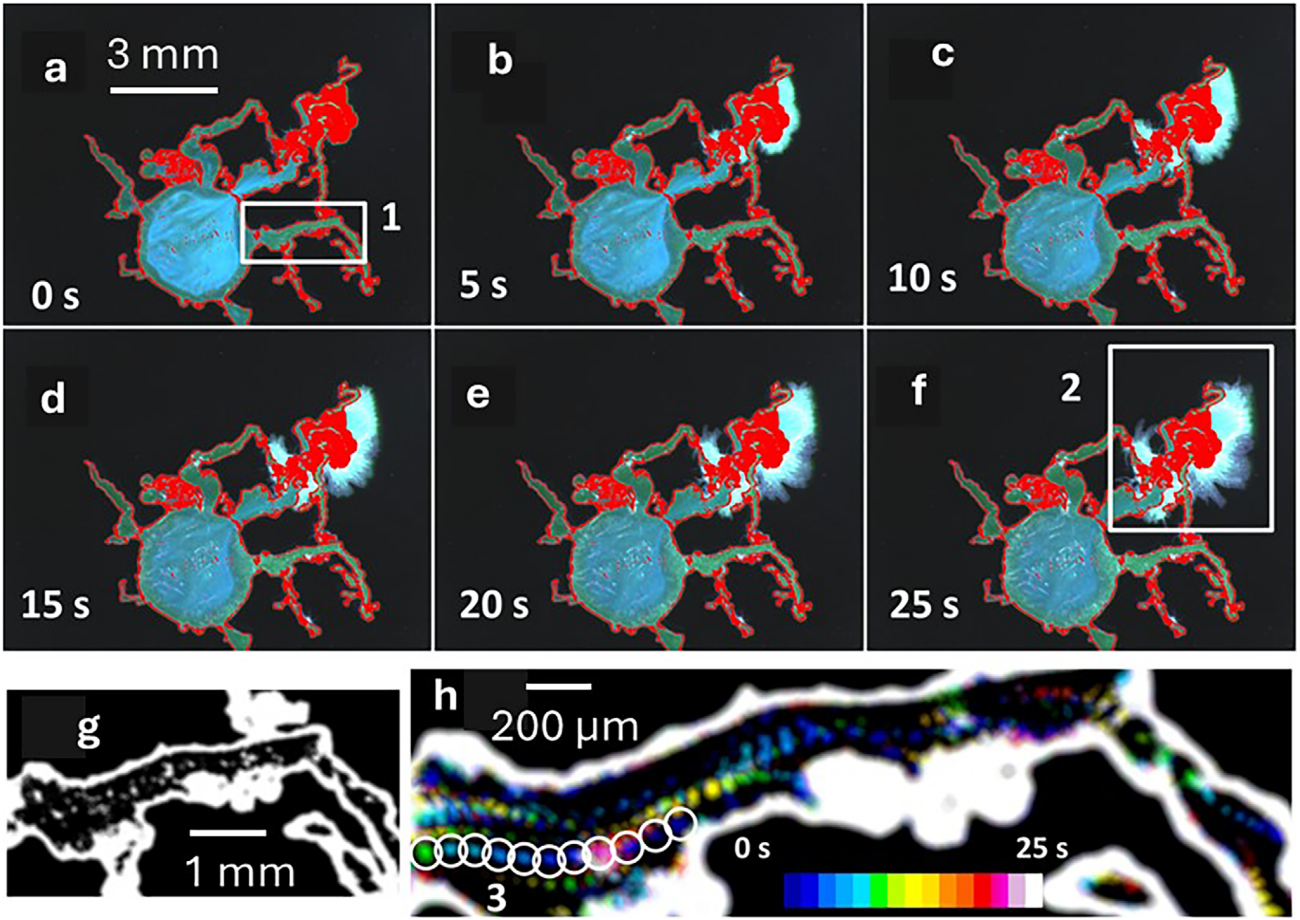
(a–f) time‐lapse images of a growing silica garden taken at 5 5‐second intervals. The initially formed membrane is shown in red. Growth occurs only in the region labelled as 2 in panel f. g) shows the particles formed in branch 1 of figure a because of the high local pH.

Figure 3a–f show the time evolution of the experiment. The initially formed membrane is displayed in red to allow an immediate visualization of the growing parts of the structure. This sequence shows that growth occurs only in the region labelled as 2 in Figure 3f. In particular, a 2D bundle of micrometric tubes erupts from this branch. It is very interesting to observe that, in contrast with all the other branches, the one that generates the microtubes presents blue fluorescence in the inner part and an acidic pH (pH = 4.2). Hence, analyzing the fluorescence color and then the pH of the chemical garden branches, it is possible to discriminate the non‐growing (dead) branches from the growing (living) ones: in particular, living branches show acidic pH and blue fluorescence.

### 2.5 Measuring Local Changes in pH in the Silica Garden during its Growth

**FIGURE 4:**
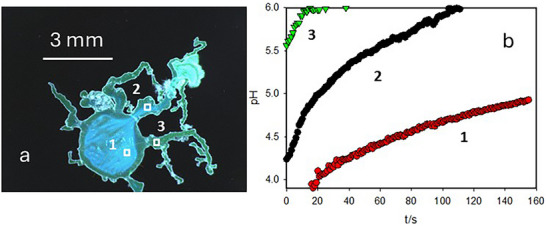
(a) RGB image of a silica garden showing three relevant regions of interest (ROI): ROI 1 is internal to the circular membrane, ROI 2 is the inner part of a living branch, and ROI 3 is the inner part of a dead branch. b) pH measured from the fluorescence in the three ROI of a).

Time evolution of pH was analyzed into three characteristic regions of interest (ROI) of the silica garden shown in Figure 4a by plotting the pH as a function of time. Inside the metal solution droplet (ROI 1 in Figure 4a), the acidic starting pH (≈4.0) increased only moderately during the microtube formation, and it reached a value of 4.5–4.6 when growth terminated after 90–100 s (see Figure 4b). Similarly, the solution contained in the living branch (ROI 2 in Figure 4a) is quite acidic (pH 4.2) but in this case the alkalinization is faster than in ROI 1 and, interestingly, when, after 90–100 s, the pH inside the branch rises to a value as high as 5.8–5.9 the microtubular structure stops growing (Figure 4b). We can hence conclude that injection of acidic MS from the living branch into the SS is at the basis of the microtube formation and that this process is paralleled by a progressive diffusion of the hydroxyl ions through the membrane, including the one of the living branch, that leads to the progressive deactivation of the branch itself. This behavior can be understood considering the pH‐dependent solubility of manganese that decreases upon rising pH as it can be observed inside the “dead” branch (ROI 3 in Figure 4a), where the initially high pH (5.5) further increases during time exceeding the upper working limit of the probe pH = 5.9 (Figure 4b).

### 2.6 Particle Formation in the Branches of Silica Gardens

This relatively high pH present in the dead branch labeled as 1 in Figure 3a (pH>5.5) leads to the precipitation of the metal in the form of microparticles that scatter illumination light and can be clearly detected by analyzing the red component of the image, not affected by fluorescence, as shown in Figure 3g. Very interestingly, the trafficking of these particles allows tracking the flows of solution in the silica garden. Starting from the time‐lapse sequence, it was possible to plot the trajectories of the particles in a single image (3 h) using a time‐coded scale where different colors correspond to different times. For the sake of clarity, the position of a selected particle at different times is further evidenced in white circles in Figure 3h: each single displacement occurs in one second, and the velocity of the particles can be calculated. It is important to note that because of the relatively large size of the particles and the scale of the observation, any diffusional contribution to their movement can be ruled out. Hence, particles are transported by the flow of the solution in the “dead” branch, and they can be used to track the flow itself.

### 2.7 Solution Flows in the Silica Gardens

It is interesting to observe in Figure 3h that all the particles move in the same direction (right to left) toward the cavity formed by the metal solution: the velocity of the particles is as high as 0.10±0.01 mm/s, and it can be used to calculate a flow rate of 3.8 pL/s.

**FIGURE 5:**
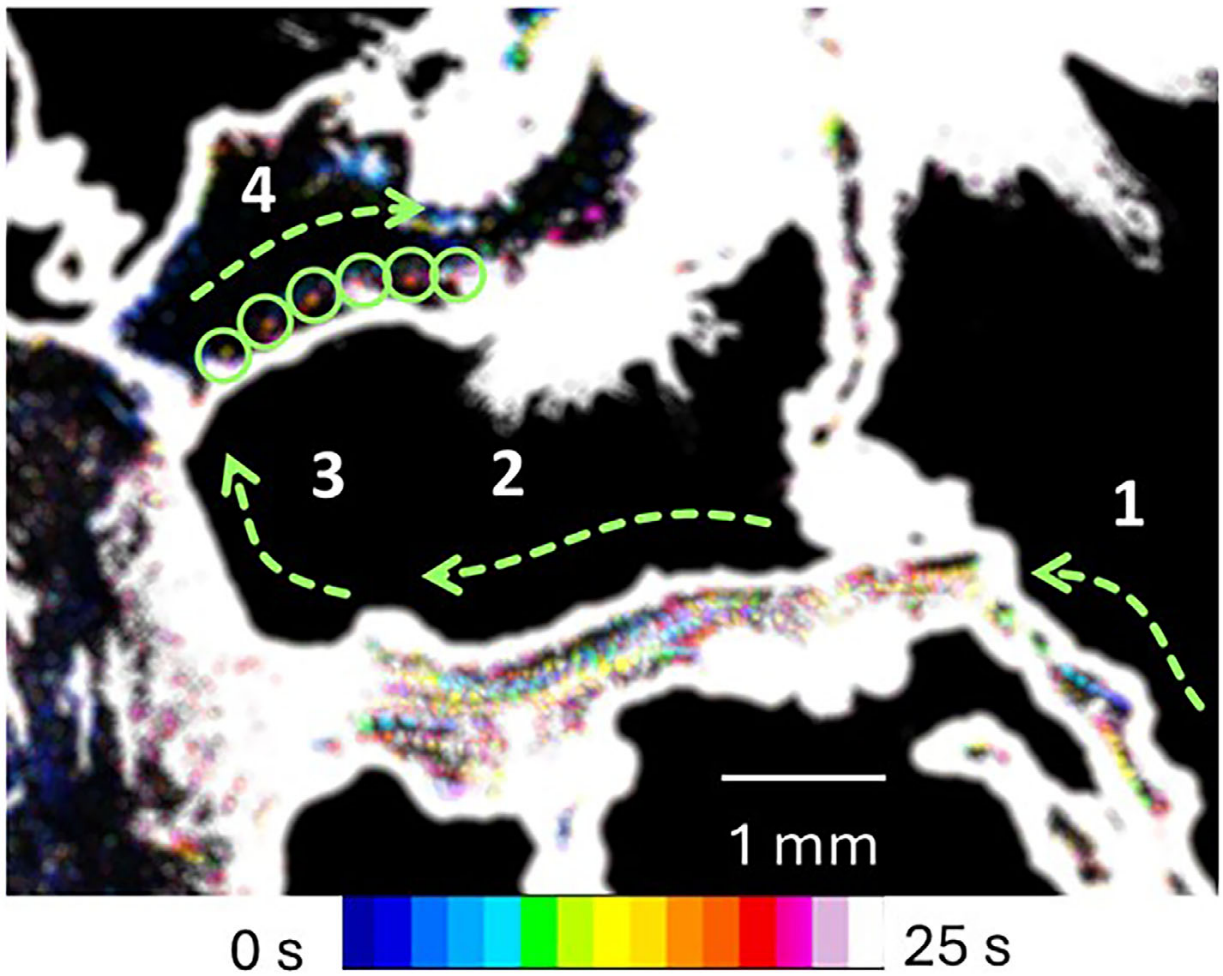
Particle trafficking inside the silica garden. A time‐related color code is used, different colors correspond to different times, as shown in the scale bar. Arrows indicate the direction of the movement of the particles over time. Circles indicate the position of a particle moving in the living branch.

A more extended view of particle trafficking inside the garden that includes a dead branch and a living branch simultaneously is given in Figure 5. Following the time‐coded scale it becomes clear that particles formed in the dead branch travels toward the main cavity (arrows 1 and 2 were added for clarity in Figure 5) and then they move in a layer of solution parallel to the membrane wall toward the living branch (arrow 3) and, finally, particles reach in the living branch as shown by arrow 4. Particle tracking clearly proves the circulation of the MS in the silica garden, and it definitely demonstrates that the MS is pumped from the dead branches into the living ones and hence into the silicate solution to produce the microtubes, but also that this process contributes to the gradual alkalinization of the solution in the living branch, leading to its “death”.

Going more into the detail, the flow rate in the living branch is 14 pL/s and hence higher than the one measured in the single examined dead branch. This observation demonstrates that solution is pumped also from other parts of the silica garden structure, and the fact that these additional flows are not evidenced by particle formation (which occurs at relatively high pH) and transportation is consistent with the injection of a fraction of quite acidic solution into the living branch.

### 2.8 Membrane Permeability and Thickening of the Silica Gardens

Distribution of the pH in the silica gardens shows an initial alkalinization of the metal ion solution in the proximity of the membrane. This suggests that the membrane is initially permeable to the external OH^−^ ions [16]. Nevertheless, mineralization, with the precipitation of Mn^2+,^ is expected to lead to an increase in pH.

As mentioned, by comparing the time‐lapsed images acquired during the silica garden growth, we could observe that, after about 90–100 s, propagation of the structure and in particular of the microtubes stopped while the thickening of the membrane continued [44]. Formation of the membrane, on the other hand, has been recently demonstrated to be controlled by diffusion [45]. It is interesting to observe in Figure 4b that the stop of the growth is concomitant with the alkalinization of the living branch solution, where the pH has risen from the initial value of 4.2 to about 5.9.

A possible explanation for this effect is that the permeability of the membrane is time‐dependent and progressively decreases with time, and the permeation rate of OH^−^ ions through the membrane also decreases with time.

In order to estimate if the width of the initial green fluorescence band in Figure 2a could be compatible with a purely diffusional process, we considered diffusion of ions and, in particular, of OH^−^ through the membrane to calculate the time‐dependent diffusion length x,:

(1) $$x\;=0.954\sqrt{Dt}$$

Hence, considering for OH^−^ a diffusional coefficient D = 5.270 10^−9^ m^2^/s [46], it was possible to estimate a displacement of 73 µm t^1/2,^ hence about 160 µm after the time t = 5 s. This value is smaller than the observed band width, suggesting that i) in addition to diffusion, other processes accelerate the penetration of alkaline ions through the membrane and hence that osmotic pressure plays a relevant role, and ii) other processes, such as mineralization, are involved in the pH change. As shown in Figure 3, the width of the less acidic (green) band increases with time as expected for a progressive alkalinization process.

### 2.9 Laser Confocal Scanning Microscope (LCSM) Analysis of the Silica Gardens

Results reported and discussed above demonstrate that the use of the pH probe HPTS and of the RGB camera in the wide‐field mode offered a very detailed description of the dynamics of the evolution of the whole silica garden. Nevertheless, this technique does not present high enough resolution, especially along the z‐axis, to give information about the morphology of the microtubes and the pH distribution inside these microstructures. LCSM was hence used to investigate in detail these features, in order to complete the scenario. We would like to stress that, on the other hand, LCSM does not allow a complete analysis of the silica garden, and it is complementary to CMOS camera detection. The optical transmission image and the confocal fluorescence image of the growing microtubes in the silica garden were acquired simultaneously in a time‐lapse experiment using the same pH probe and experimental preparative approach described for wide‐field microscopy, also in this case, the fluorescence was acquired into two separate channels: blue and green. LCSM allows scanning relatively small areas, and it is not suitable to image the whole silica garden, so after spotting the growing microtubes, an area of 1417×1417 µm^2^ was analyzed.

**FIGURE 6:**
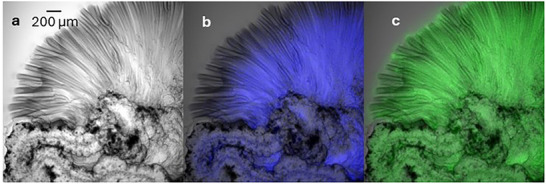
Confocal microscope image of growing microtubes. a) optical transmittance, b) blue fluorescence, c) green fluorescence.

The first acquired transmission optical image of growing microtubes is shown in Figure 6a, while the corresponding fluorescence B and G images are shown in blue and green Figure 6b,c respectively. It is interesting to notice that the two fluorescence signals show a very different spatial distribution. In particular, the blue signal decreases toward the proximity of the microtube tips while the green signal is quite intense in the top area. Considering that the B fluorescence is more intense at less acidic pH while the G contribution increases at higher pH it is possible to conclude that pH in the proximity of the tubes terminations is higher with respect to the one detected in the inner section.

**FIGURE 7:**
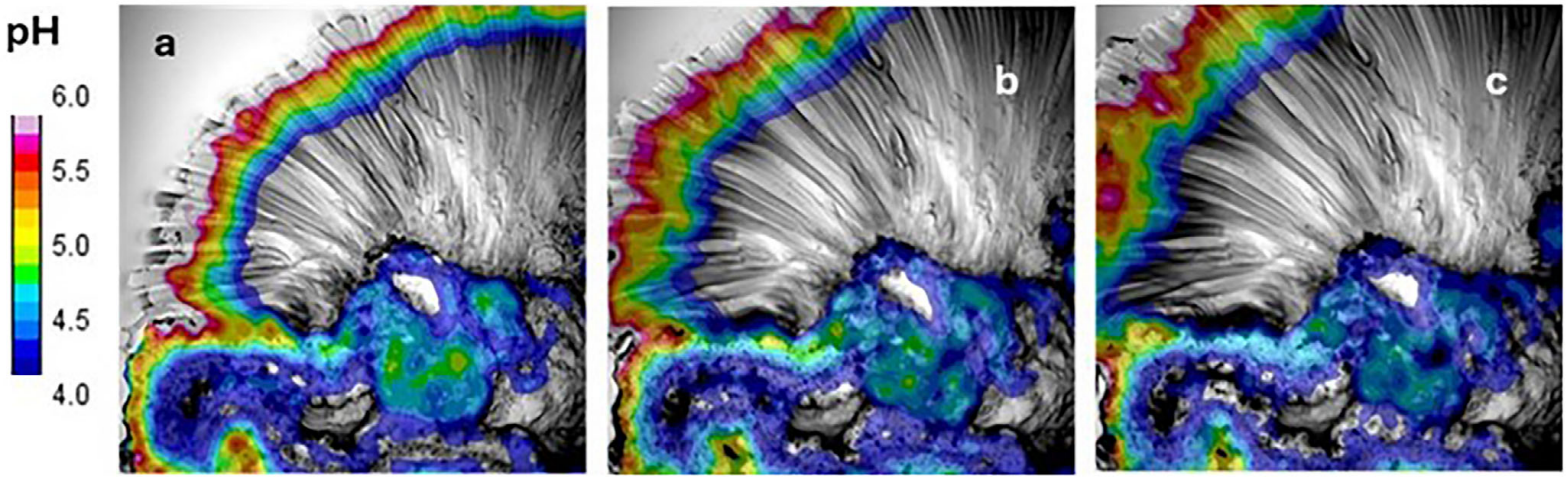
Time lapsed pH maps (in false color) calculated by ratiometric analysis at time intervals of 5 second. a) pH map at 0 s, b) pH map at 5 s, c) pH map ar 10 s.

In order to investigate the pH distribution with higher resolution, the same ratiometric approach described for wide filed microscopy was followed by calculating the map of the intensity ratio and hence, the local value of the pH. In Figure 7 these calculated values were represented into false colors and overlapped to the transmission optical image in order to correlate pH distribution to the morphology of the tubes at different time of growth hence at the beginning of the observation (0 s, Figure 7a) and after 5 s and 10 s (Figure 7b,c respectively).

Strong green fluorescence on the contrary is detected on the tips of the microtubes (Figure 6c) but because, on the contrary the corresponding blue emission is rather weak calculation of the intensity ratio just indicates that the pH in this region (marked in Figure 7 as a white band) is above the upper working limit of the probe hence pH >5.9. Figure 7,a clearly reveals that moving longitudinally from the tips inside the microtubes the pH gradually decreases quite linearly with the distance down to values below 3.9, moreover this two unit pH drop occurs in a band about 180 µm broad hence with a pH gradient as high as 11 µm^−1^.

Additionally, Figure 7b,c show that the observed pH pattern propagates together with the microtubes, being the pH profile across the tube and with respect to the tip position maintained after 5s (Figure 7b) and 10s (Figure 7c).

Fluorescence LCSM results demonstrate for the first time that huge longitudinal pH gradients are generated inside the microtubes of the silica garden, giving a new vision of the mechanism of growth of self‐assembled inorganic structures.

## 3 Conclusions

Silica garden membrane growth has been visualized in situ, in real time, using a fluorescent ratiometric method with HPTS fluorescent dye. The very intense fluorescence signal in the blue and the green regions is also pH‐dependent, while fluorescence in the red is negligible. Therefore, the blue and green components measure only fluorescence intensity, while the red component measures only scattering intensity. This allows the use of an RGB camera for wide field time‐lapsed imaging to follow the pH distribution and evolution, as well as the precipitation of the pattern and movement of particles, and to track fluid flow within the garden.

During the formation of the membrane, alkalinization of the metal solution occurs because of the permeation of OH^−^ ions through the membrane. The overall pH in the initial metal solution, the living branch, and the dead branch is distinctively different. The pH value increases from the metal solution to live branches and dead branches, and the rate of pH change rises in the same sequence.

The same ratiometric method has been applied to fluorescence laser confocal scanning microscopy to analyze a 2D bundle of micrometric tubes emerging from this branch. This has demonstrated a longitudinal increase in pH towards the tips of the tubes during their growth, and very large pH gradients within the microtubules.

Particle formation was also observed in the wide‐field images. Tracking the motion of these particles reveals, for the first time, fluid flow dynamics in silica gardens. The average flow velocity is 0.10 ± 0.01 mm/s, corresponding to a flow rate of 3.8 pL/s.

## 4 Experimental Section

### 4.1 Preparation of Silica Gardens

A saturated solution of MnSO_4_ was prepared by stirring for 2 hours, 10 mL of ultrapure water (Milli‐Q) with 12 g of Manganese (II) sulfate monohydrate by Aldrich. After 24 hours, the non‐solubilized solid was precipitated, and the transparent pink solution was used for the experiments. In order to detect pH‐dependent fluorescence, 8‐Hydroxypyrene‐1,3,6‐trisulfonic acid trisodium salt (HPTS) by Aldrich was added. A Silicate solution was prepared by mixing commercial Sodium Metasilicate by Aldrich with water in a 1:2 volume ratio. In a typical experiment, 2 µL of metal‐saturated solution was placed in the center of a microscope cover‐glass (24×40 mm, about 0.1 mm thick) while 20 µL of the silicate solution was placed on a microscope glass (75×25 mm, about 1 mm thick). Two strips of double‐sided adhesive tape (20×3 mm, about 0.1 mm thick) were placed on the microscope glass at about 34 mm from each other. The cover‐glass was gently turned upside down, and the cell was closed by immersing the metal solution in the silica solution. The cell was hence positioned in the observation stage, and the images were detected ten seconds after cell preparation. The methodology used in this paper was very similar to the one we used in [4]. In the work reported in that paper, we poured microdrops of metal solutions into sodium silicate solutions. Among the salts we used was MnCl_2_. We found that the membranes were formed by For MnCl_2_ membranes, the identity phases were Hausmannite Mn_3_O_4_, Kempite Mn_2_Cl(OH)_3_, Pyrochroite Mn(OH)_2_.

### 4.2 Optical Set Up

Images were acquired with a CMOS color camera DFK 23UP031 by The Imaging Source connected through USB 3.0. The camera was equipped with a 12 mm focal Computar objective. Images were acquired with the software IC Capture 2.5 and processed with the software Image J. Irradiation was per with a LED by OSRAM with an emission peak at 365 nm.

## Conflicts of Interest

The authors declare no conflicts of interest.

## Data Availbility Statement

The data that support the findings of this study are available in the supplementary material of this article.

## Supporting information




**Supporting file 1**: smll72040‐sup‐0001‐SuppMat.docx.

**Supporting file 2**: smll72040‐sup‐0002‐VideoS1.avi.

**Supporting file 3**: smll72040‐sup‐0003‐VideoS2.avi.

**Supporting file 4**: smll72040‐sup‐0004‐VideoS3.avi.

**Supporting file 5**: smll72040‐sup‐0005‐VideoS4.avi.

**Supporting file 6**: smll72040‐sup‐0006‐VideoS5.avi.

**Supporting file 7**: smll72040‐sup‐0007‐VideoS6.avi.

**Supporting file 8**: smll72040‐sup‐0008‐VideoS7.avi.

**Supporting file 9**: smll72040‐sup‐0009‐VideoS8.avi.

**Supporting file 10**: smll72040‐sup‐0010‐VideoS9.avi.

**Supporting file 11**: smll72040‐sup‐0011‐VideoS10.avi.

**Supporting file 12**: smll72040‐sup‐0012‐VideoS11.avi.

**Supporting file 13**: smll72040‐sup‐0013‐VideoS12.avi.

**Supporting file 14**: smll72040‐sup‐0014‐VideoS13.avi.

**Supporting file 15**: smll72040‐sup‐0015‐VideoS14.avi.

**Supporting file 16**: smll72040‐sup‐0016‐VideoS15.avi.

**Supporting file 17**: smll72040‐sup‐0017‐VideoS16.avi.
