# Supplementary material for: In Situ, Real‐Time Imaging of pH Gradients and Solution Flows through Silica Garden Membranes during Tube Growth
Source: Small. 2025 Dec 22;22(9):e07110. doi: 10.1002/smll.202507110 (PMC12895228; doi:10.1002/smll.202507110)
Supplement: Supplementary file 1 — Supporting file 1: smll72040‐sup‐0001‐SuppMat.docx. [file SMLL-22-e07110-s004.docx]

Supporting Information

In-situ, real-time imaging of pH gradients and solution flows through silica garden membranes during tube growth

Arianna Menichetti, Jeannette Manzi, Dario Mordini, Fermín Otálora, Sara Moreno San Juan, Juan Manuel García-Ruiz* and Marco Montalti*

**Optical setup**

Images were acquired with a CMOS color camera DFK 23UP031 by The Imaging Source connected through USB 3.0. The camera was equipped with a 12 mm focal Computar objective. Images were acquired with the software IC Capture 2.5 and processed with the software Image J. Images were acquired in reflectance mode. Hence, as schematized in Figure S1 the irradiation and the detection were performed on the same side.

The camera sensor is Aptina MT9P031(<https://www.onsemi.com/pdf/datasheet/mt9p031-d.pdf>). The sensor is made by 2592 x 1944 pixel. Pixel size is 2.2 x 2.2 µm and the total size of the sensor is 5.70 x 4.28 mm.


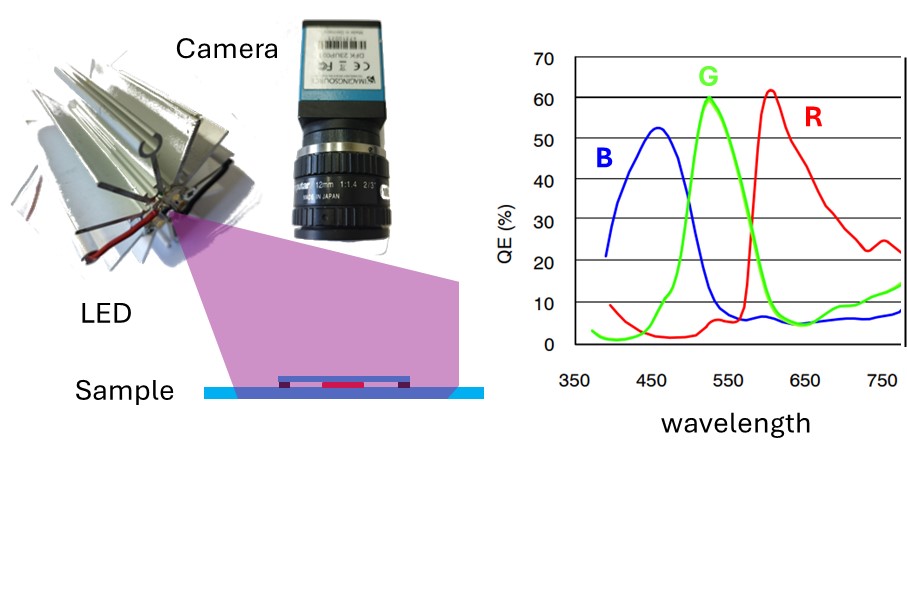


**Figure S1**. Left: scheme of the optical setup used for image acquisition. The sample was illuminated with a LED with peak emission at 365 nm and images were acquired with a color camera. Right: response spectra of the R, G and B sensors of the camera.


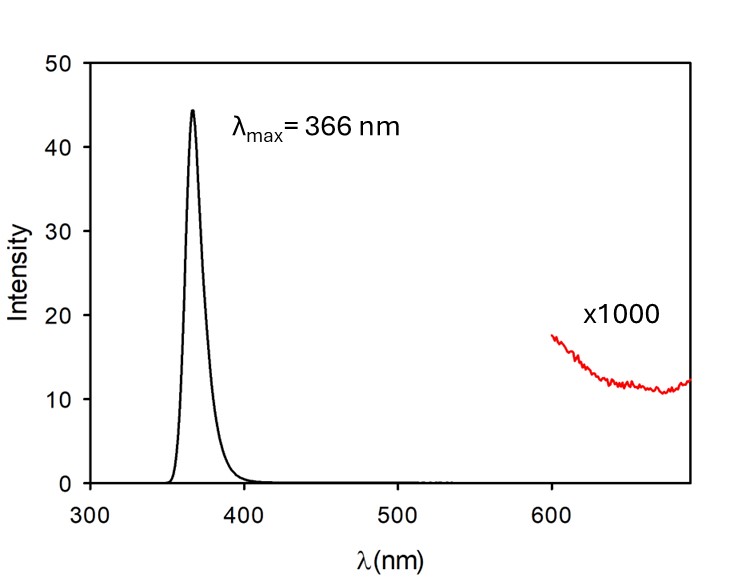
Choosing as detection window for three channel RGB the wavelengths at which the QE(%) is 50% of the maximum the B channel corresponds to 400 nm < λ < 500 nm, G to 500 nm < λ < 600 nm

**Figure S2**. Emission spectrum of the LED used for excitation (black line). The LED presents a weak emission in the red (red curve).

**Effect of the addition of NaOH to a saturated MnSO_4_ water solution**

In order to model the effect of the penetration of an alkaline solution through the silica garden membrane a concentrated solution of NaOH (4.6 mM) was added to 5 mL of a saturated solution of MnSO_4_. Changes in the pH of the solution as a function of the concentration of NaOH are shown in Figure S3.

As shown in Figure S3 the pH measured upon addition of NaOH in the presence of saturated MnSO_4_ (black dots) is more acidic than expected in the case of the same addition to pure water (red squares) demonstrating that MnSO_4_ works as a buffer. The pH data were fitted according to the equation shown in Figure S3.


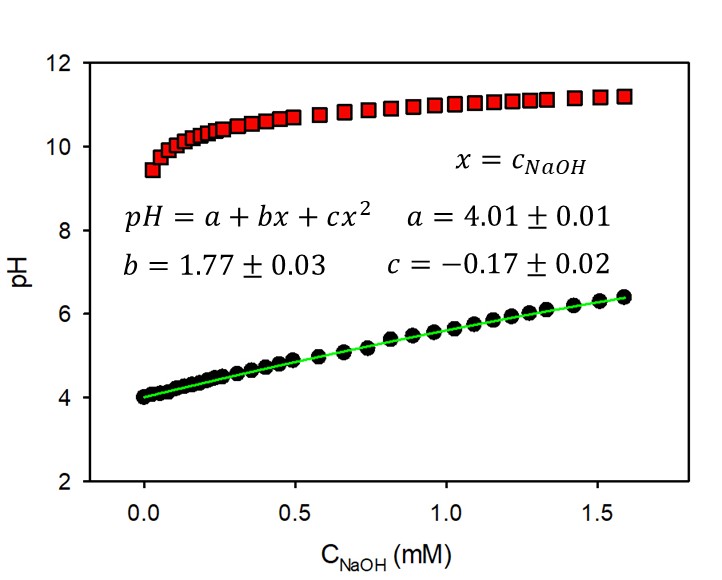
**Figure S3**. pH of a saturated water solution of MnSO_4_ upon addition of NaOH (black dots). Measured pH is considerably lower than expected for NaOH in pure water (red squares).

Fluorescence spectra as a function of pH

Fluorescence spectra of a saturated water solution of MnSO_4_ containing HPTS 0.1 mM at different pH, at 365 nm excitation, are shown in Figure S4.


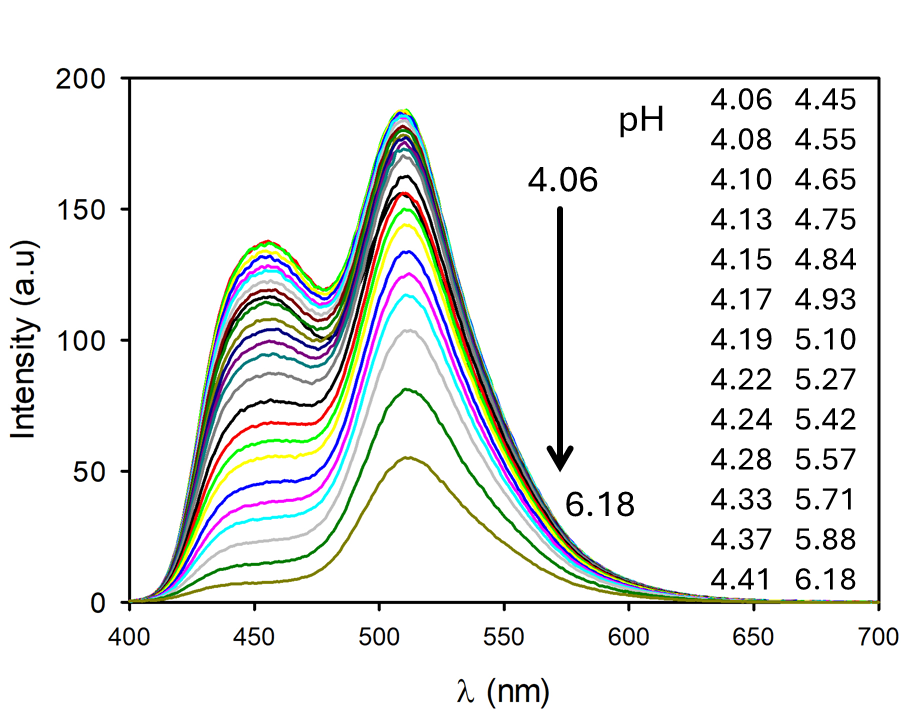
Figure S4. Fluorescence spectra of a saturated water solution of MnSO_4_ containing HPTS 0.1 mM upon excitation at 365 nm. Addition of a concentrated solution of NaOH (4.6 mM) allowed to increase the initial pH to the values shown in the figure.

Considering the spectra of Figure S4 and the spectral response of Figure S1 the two component G and B were calculated, and the ratio was plotted in Figure S5. As shown in Figure S5 the ratio R could be fitted with a simple monoprotic model (red curve) where R_1_ is R at pH <<pK_a_ and R_2_ is R at pH >> pK_a_ with an apparent pK_a_=4.81±0.03.


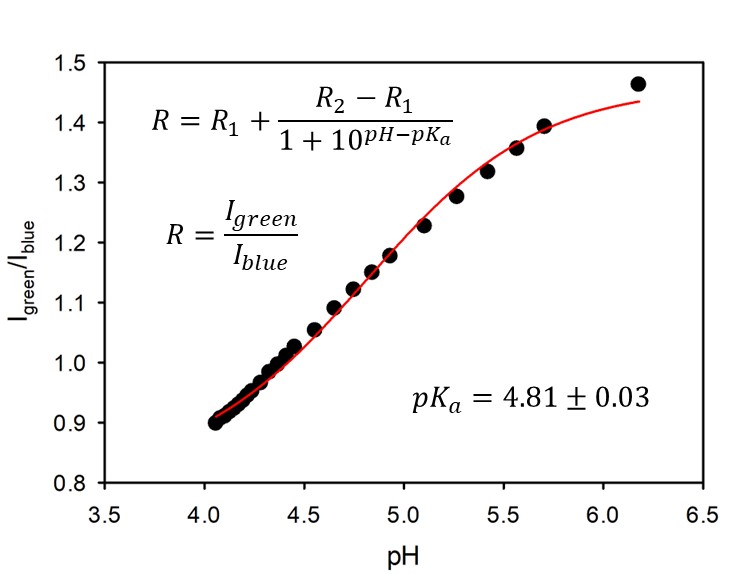


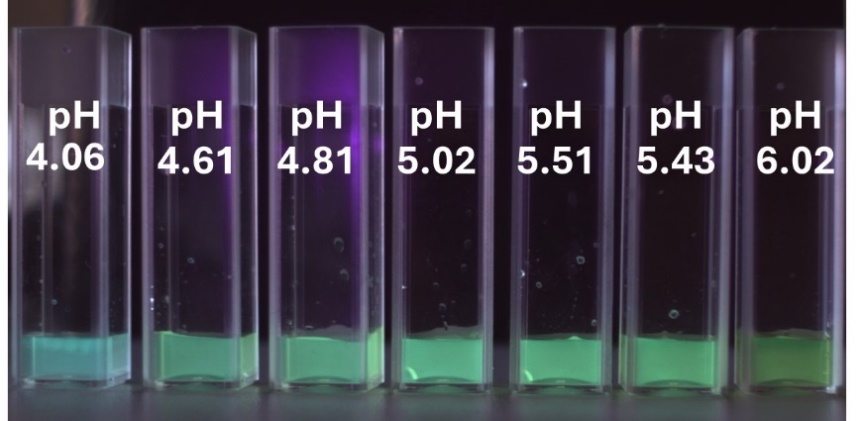
Figure S5. Ratio between the intensity of the green (G) fluorescence and of the blue fluorescence, calculated from the spectra in Figure S4 using the spectral response curves of Figure S1. Data could be fitted with a simple monoprotic model giving and apparent pK_a_ = 4.81±0.03.

Figure S6. Fluorescence image of seven cuvettes containing a MnSO_4_ saturated water solution and HPTS 0.1 mM at increasing pH. pH was adjusted by adding NaOH. The cuvette on the left contains the metal solution without any added base.

Fluorescence images calibration

In order to convert the fluorescence ratio R measured locally in time during chemical gardens growth a calibration experiment was conducted using the optical setup schematized in Figure S1. In particular, increasing amounts of NaOH solution were added to a saturated solution of MnSO_4_ containing HPTS 0.1 mM. Seven different cuvettes with the pH showed in Figure S6 were prepared and an image was acquired under excitation. The picture clearly shows the change of the fluorescence color from cyan to green upon pH increase.


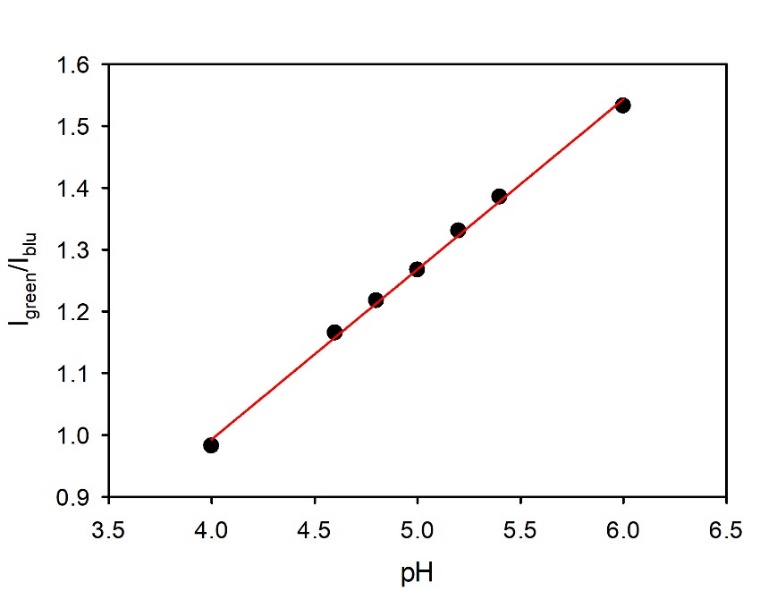
Image of Figure S6 was processed with the software Image J to get the B and G component. Hence the G/B intensity ratio was plotted in Figure S7 and fitted linearly to convert the intensity ratio measured during the silica garden growth into pH.

Figure S7. Ratio of the green component intensity and blue component intensity measured in the image of Figure S6.

LCSM images acquisition

Time lapsed images were acquired with LCSM Zeiss Axio Imager A1 Microscope

pH measurements

Bulk pH measurements were performed using a bench pH-meter by Violab equipped with a semi-micro pH electrode.

Effect of other metal ions on HPTS fluorescence

In order to test the applicability of HTPS to measure pH in concentrated solutions of metal salts different from MnSO_4_ we compared optical and fluorescence images of saturated solution MnSO_4_ of CrCl_3_, CoCl_2_ and FeCl_3_ containing HPTS 0.1 mM. Images are shown in Figure S7 and they clearly show that only the solution containing manganese is poorly colored (Figure S8a). Moreover, upon UV excitation (365 nm) only the manganese solution shows an intense blue fluorescence while the pH probe is quenched by the other metal ions.


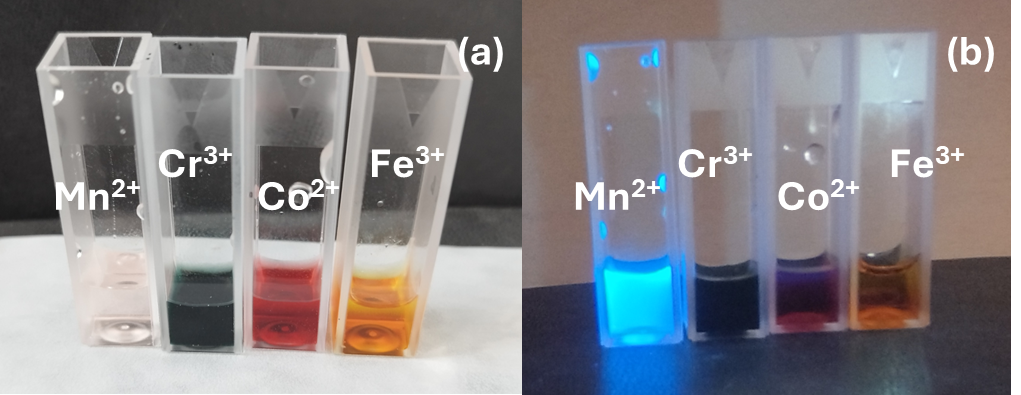
Figure S8. a) optical images, under ambient illumination, of saturated solution of MnSO_4_ of CrCl_3_, CoCl_2_ and FeCl_3_.b) Fluorescence image under irradiation with 365 nm LED.


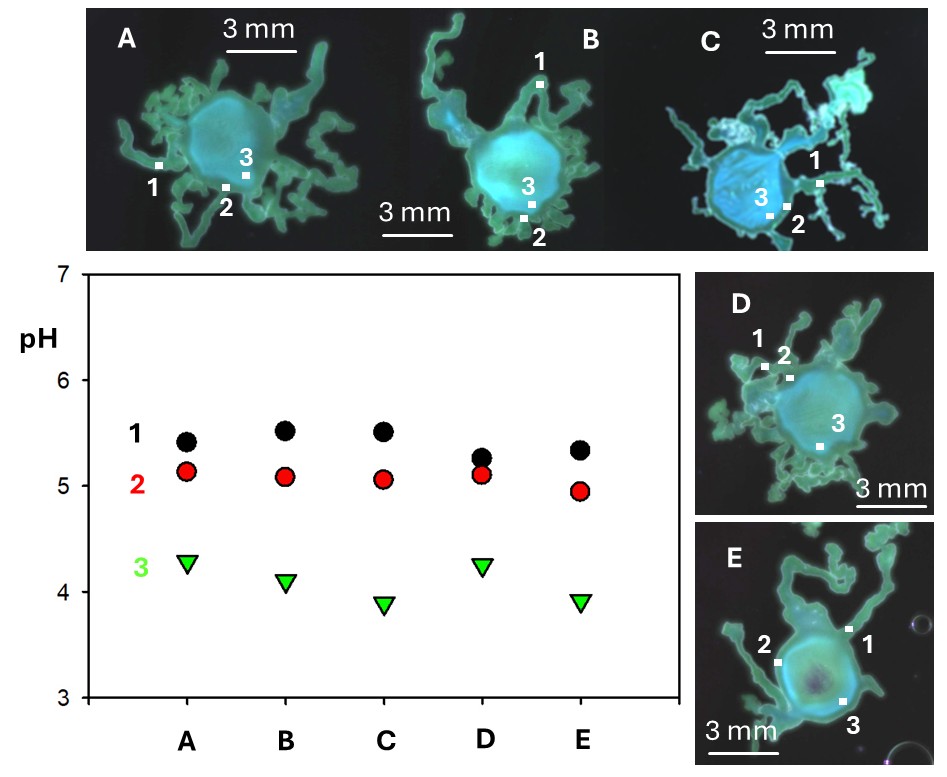


Figure S8. (A-E) images of five silica gardens prepared as reported in the experimental section of the paper. Fluorescence of HPTS was detected using the setup described above. G/B fluorescence ratio was used to calculate pH in three relevant positions of the gardens: one dead branch (1), in metal solution in proximity of the circular membrane (2) and in the inner part of the metal solution.

Reproducibility of the results

Results reported in the main paper are given for a specific silica garden. Each silica garden growth is a unique experiment, and final morphology cannot be predicted and is expected to be different in every case. Nevertheless, in order to demonstrate that main features of the experiment are reproducible we analyzed five different cases of growth of silica garden in the same conditions discussed in the paper (further 10 videos of silica garden prepared at other silicate concentrations conditions are also available as supporting information). As shown in figure S9 all the five silica gardens (labelled as A-E) present a circular central part, where the initial metal ion solution was placed, and some branches. Some branches are closed and do not grow (as shown in the videos). The pH in these dead branches (in the point labelled as 1) is relatively high as shown in figure S9. A band of metal solution with relatively high pH (measured in the point labelled as 2) was observed in the proximity of the central circular membrane. The inner part of the metal solution, on the other hand, was, in the five cases more acidic, as demonstrated by the pH measured in the point labelled as 3 in figure S9, where the pH is, in all the five cases, close to 4.0.

References

1. García-Ruiz, J. M.; Otálora, F., 1 - Crystal Growth in Geology: Patterns on the Rocks. In *Handbook of Crystal Growth (Second Edition)*, Rudolph, P., Ed. Elsevier: Boston, 2015; pp 1-43.

2. Nakouzi, E.; Steinbock, O., *Science Advances 2* (8), e1601144. DOI 10.1126/sciadv.1601144.

3. Cera, L.; Schalley, C. A., *Advanced Materials* **2018,** *30* (38), 1707029. DOI <https://doi.org/10.1002/adma.201707029>.

4. Mattia Bizzarri, B.; Botta, L.; Pérez-Valverde, M. I.; Saladino, R.; Di Mauro, E.; García-Ruiz, J. M., **2018,** *24* (32), 8126-8132. DOI <https://doi.org/10.1002/chem.201706162>.

5. Opel, J.; Brunner, J.; Zimmermanns, R.; Steegmans, T.; Sturm, E.; Kellermeier, M.; Cölfen, H.; García-Ruiz, J.-M., **2019,** *29* (37), 1902047. DOI <https://doi.org/10.1002/adfm.201902047>.

6. Zhang, G.; Verdugo-Escamilla, C.; Choquesillo-Lazarte, D.; García-Ruiz, J. M., *Nature Communications* **2018,** *9* (1), 5221. DOI 10.1038/s41467-018-07658-0.

7. Noorduin, W. L.; Grinthal, A.; Mahadevan, L.; Aizenberg, J., **2013,** *340* (6134), 832-837. DOI doi:10.1126/science.1234621.

8. Holtus, T.; Helmbrecht, L.; Hendrikse, H. C.; Baglai, I.; Meuret, S.; Adhyaksa, G. W. P.; Garnett, E. C.; Noorduin, W. L., *Nature Chemistry* **2018,** *10* (7), 740-745. DOI 10.1038/s41557-018-0064-1.

9. Getenet, M.; García-Ruiz, J. M.; Verdugo-Escamilla, C.; Guerra-Tschuschke, I., **2020,** *10* (6), 467.

10. García-Ruiz, J. M.; Nakouzi, E.; Kotopoulou, E.; Tamborrino, L.; Steinbock, O., **2017,** *3* (3), e1602285. DOI doi:10.1126/sciadv.1602285.

11. Cardoso, S. S. S.; Cartwright, J. H. E.; Sainz-Díaz, C. I., *Icarus* **2019,** *319*, 337-348. DOI <https://doi.org/10.1016/j.icarus.2018.09.020>.

12. García-Ruiz, J. M.; van Zuilen, M. A.; Bach, W., *Physics of Life Reviews* **2020,** *34-35*, 62-82. DOI <https://doi.org/10.1016/j.plrev.2020.01.001>.

13. Kellermeier, M.; Glaab, F.; Melero-García, E.; García-Ruiz, J. M., *Methods in enzymology* **2013,** *532*, 225-56. DOI 10.1016/b978-0-12-416617-2.00011-4.

14. Barge, L. M.; Cardoso, S. S. S.; Cartwright, J. H. E.; Cooper, G. J. T.; Cronin, L.; De Wit, A.; Doloboff, I. J.; Escribano, B.; Goldstein, R. E.; Haudin, F.; Jones, D. E. H.; Mackay, A. L.; Maselko, J.; Pagano, J. J.; Pantaleone, J.; Russell, M. J.; Sainz-Díaz, C. I.; Steinbock, O.; Stone, D. A.; Tanimoto, Y.; Thomas, N. L., *Chemical Reviews* **2015,** *115* (16), 8652-8703. DOI 10.1021/acs.chemrev.5b00014.

15. Getenet, M.; Rieder, J.; Kellermeier, M.; Kunz, W.; Manuel García-Ruiz, J., *Chemistry – A European Journal* **2021,** *27* (65), 16135-16144. DOI <https://doi.org/10.1002/chem.202101417>.

16. Glaab, F.; Kellermeier, M.; Kunz, W.; Morallon, E.; García-Ruiz, J. M., *Angewandte Chemie International Edition* **2012,** *51* (18), 4317-4321. DOI <https://doi.org/10.1002/anie.201107754>.

17. Chin, K.; Pasalic, J.; Hermis, N.; Barge, L. M., **2020,** *85* (12), 2619-2628. DOI <https://doi.org/10.1002/cplu.202000600>.

18. Saladino, R.; Botta, G.; Bizzarri, B. M.; Di Mauro, E.; Garcia Ruiz, J. M., *Biochemistry* **2016,** *55* (19), 2806-2811. DOI 10.1021/acs.biochem.6b00255.

19. Saladino, R.; Di Mauro, E.; García-Ruiz, J. M., **2019,** *25* (13), 3181-3189. DOI <https://doi.org/10.1002/chem.201803889>.

20. Thouvenel-Romans, S.; Saarloos, W. v.; Steinbock, O., *Europhysics Letters* **2004,** *67* (1), 42. DOI 10.1209/epl/i2003-10279-7.

21. Pagano, J. J.; Bánsági, T.; Steinbock, O., *The Journal of Physical Chemistry C* **2007,** *111* (26), 9324-9329. DOI 10.1021/jp071534q.

22. Glaab, F.; Rieder, J.; Klein, R.; Choquesillo-Lazarte, D.; Melero-Garcia, E.; García-Ruiz, J.-M.; Kunz, W.; Kellermeier, M., *ChemPhysChem* **2017,** *18* (4), 338-345. DOI <https://doi.org/10.1002/cphc.201600748>.

23. Hussein, S.; Maselko, J.; Pantaleone, J. T., *Langmuir* **2016,** *32* (3), 706-711. DOI 10.1021/acs.langmuir.5b04196.

24. Farkas, P. E.; Lantos, E.; Horváth, D.; Tóth, A., **2024,** *6* (6), e202400053. DOI <https://doi.org/10.1002/syst.202400053>.

25. Balog, E.; Bittmann, K.; Schwarzenberger, K.; Eckert, K.; De Wit, A.; Schuszter, G., *Physical Chemistry Chemical Physics* **2019,** *21* (6), 2910-2918. DOI 10.1039/C8CP07693F.

26. Thouvenel-Romans, S.; Pagano, J. J.; Steinbock, O., *Physical Chemistry Chemical Physics* **2005,** *7* (13), 2610-2615. DOI 10.1039/B504407C.

27. Zhao, W.; Sakurai, K., *ACS Omega* **2017,** *2* (8), 4363-4369. DOI 10.1021/acsomega.7b00930.

28. Satoh, H.; Tsukamoto, K.; Garcia-Ruiz, J. M., *European Journal of Mineralogy* **2014,** *26* (3), 415-426. DOI 10.1127/0935-1221/2014/0026-2378.

29. Glaab, F.; Rieder, J.; García-Ruiz, J. M.; Kunz, W.; Kellermeier, M., *Physical Chemistry Chemical Physics* **2016,** *18* (36), 24850-24858. DOI 10.1039/C6CP02107G.

30. Nagatsu, Y.; Ishii, Y.; Tada, Y.; De Wit, A., *Physical Review Letters* **2014,** *113* (2), 024502. DOI 10.1103/PhysRevLett.113.024502.

31. Montalti, M.; Zhang, G.; Genovese, D.; Morales, J.; Kellermeier, M.; García-Ruiz, J. M., *Nature Communications* **2017,** *8* (1), 14427. DOI 10.1038/ncomms14427.

32. Menichetti, A.; Manzi, J.; Otálora, F.; Montalti, M.; García-Ruiz, J. M., **2024,** *4* (8), 2400090. DOI <https://doi.org/10.1002/smsc.202400090>.

33. Lei, Z. H.; Zhang, F., *Angewandte Chemie-International Edition* **2021,** *60* (30), 16294-16308. DOI 10.1002/anie.202007040.

34. Feng, Z.; Tang, T.; Wu, T.; Yu, X.; Zhang, Y.; Wang, M.; Zheng, J.; Ying, Y.; Chen, S.; Zhou, J.; Fan, X.; Zhang, D.; Li, S.; Zhang, M.; Qian, J., *Light: Science & Applications* **2021,** *10* (1), 197. DOI 10.1038/s41377-021-00628-0.

35. Yin, J.; Huang, L.; Wu, L.; Li, J.; James, T. D.; Lin, W., *Chemical Society Reviews* **2021,** *50* (21), 12098-12150. DOI 10.1039/d1cs00645b.

36. Mancini, F.; Menichetti, A.; Degli Esposti, L.; Montesi, M.; Panseri, S.; Bassi, G.; Montalti, M.; Lazzarini, L.; Adamiano, A.; Iafisco, M., **2023,** *14* (2), 90.

37. Mavridi-Printezi, A.; Giordani, S.; Menichetti, A.; Mordini, D.; Zattoni, A.; Roda, B.; Ferrazzano, L.; Reschiglian, P.; Marassi, V.; Montalti, M., *Nanoscale* **2023,** *16* (1), 299-308. DOI 10.1039/d3nr04696f.

38. Zhang, J.; Chen, Y.; Wang, S.; Gong, J., *Chemical Communications* **2025,** *61* (31), 5766-5769. DOI 10.1039/D4CC06603K.

39. Montalti, M.; Prodi, L.; Zaccheroni, N., *Journal of Materials Chemistry* **2005,** *15* (27-28), 2810-2814. DOI 10.1039/B502212F.

40. Kumar, R.; Yadav, R.; Kolhe, M. A.; Bhosale, R. S.; Narayan, R., *Polymer* **2018,** *136*, 157-165. DOI <https://doi.org/10.1016/j.polymer.2017.12.056>.

41. Scarciglia, A.; Di Gregorio, E.; Aime, S.; Ferrauto, G., *Molecules (Basel, Switzerland)* **2022,** *27* (8). DOI 10.3390/molecules27082490.

42. Lee, M. H.; Kim, J. S.; Sessler, J. L., *CHEMICAL SOCIETY REVIEWS* **2015,** *44* (13), 4185-4191. DOI 10.1039/c4cs00280f.

43. Bonacchi, S.; Cantelli, A.; Battistelli, G.; Guidetti, G.; Calvaresi, M.; Manzi, J.; Gabrielli, L.; Ramadori, F.; Gambarin, A.; Mancin, F.; Montalti, M., *Angewandte Chemie-International Edition* **2016,** *55* (37), 11064-11068. DOI 10.1002/anie.201604290.

44. Wang, Q.; Bentley, M. R.; Steinbock, O., *The Journal of Physical Chemistry C* **2017,** *121* (26), 14120-14127. DOI 10.1021/acs.jpcc.7b02778.

45. Zheng, M.; Welche, P.; Cardoso, S.; Huppert, H.; Cartwright, J.; Routh, A., *Philosophical Transactions A* **2025,** *383*, 20240266. DOI 10.1098/rsta.2024.0266.

46. <https://www.aqion.de/site/diffusion-coefficients>. In [*https://www.aqion.de/site/diffusion-coefficients*](https://www.aqion.de/site/diffusion-coefficients).

**Description of the video uploaded as Supporting Information**

Video 1_1_01, 1_1_02, 1_1_03, 1_1_04, 1_1_05 were recorded using a silicate solution 1:1, V:V obtained by mixing a commercial silicate solution (see main article) and Millipore water

Video 1_2_01, 1_2_02, 1_2_03, 1_2_04, 1_2_05 were recorded using a silicate solution 1:2, V:V obtained by mixing a commercial silicate solution (see main article) and Millipore water

Video 1_3_01, 1_3_02, 1_3_03, 1_3_04, 1_3_05 were recorded using a silicate solution 1:3, V:V obtained by mixing a commercial silicate solution (see main article) and Millipore water

HTPS01 is the video analyzed in the paper
